# Contemporaneous effects of diabetes mellitus and hypothyroidism on spermatogenesis and immunolocalization of Claudin-11 inside the seminiferous tubules of mice

**DOI:** 10.1186/s12861-018-0174-4

**Published:** 2018-06-26

**Authors:** Nazar Ali KOREJO, Quanwei WEI, Kaizhi ZHENG, Dagan MAO, Rashid Ali KOREJO, Atta Hussain SHAH, Fangxiong SHI

**Affiliations:** 1 Laboratory of Animal Reproduction, College of Animal Science and Technology, Nanjing Agricultural University, Nanjing, 210095 China; 2 Faculty of Animal Husbandry and Veterinary Sciences, Sindh Agriculture University Tandojam, Hyderabad, 70060 Pakistan; 3 Department of Animal Nutrition, Faculty of Animal Production and Technology, Shaheed Benazir Bhutto University of Veterinary and Animal Sciences, Sakrand, 67210 Pakistan

**Keywords:** Diabetes, Hypothyroidism, Testis, Claudin-11, Epididymis, Spermatogenesis

## Abstract

**Background:**

Diabetes and hypothyroidism produce adverse effects on body weight and sexual maturity by inhibiting body growth and metabolism. The occurrence of diabetes is always accompanied with thyroid dysfunction. Thus, it is important to take hypo- or hyper-thyroidism into consideration when exploring the adverse effects caused by diabetes. Previous reports have found hypothyroidism inhibits testicular growth by delaying Sertoli cell differentiation and proliferation. Hence, by establishing a mouse model of diabetes combined with hypothyroidism, we provided evidence that poly glandular autoimmune syndrome affected testicular development and spermatogenesis.

**Results:**

we mimicked polyglandular deficiency syndrome in both immature and prepubertal mice by induction of diabetes and hypothyroidism, which caused decreases in serum concentrations of testosterone and insulin like growth factor 1 (IGF-1). Such reduction of growth factor resulted in inhibition of testicular and epididymal development. Moreover, expressions of Claudin-11 were observed between Sertoli cells and disrupted in the testes of syndrome group mice. We also found reduced sperm count and motility in prepubertal mice.

**Conclusions:**

This mimicry of the diabetes and thyroid dysfunction, will be helpful to better understand the reasons for male infertility in diabetic-cum-hypothyroid patients.

**Electronic supplementary material:**

The online version of this article (10.1186/s12861-018-0174-4) contains supplementary material, which is available to authorized users.

## Background

Diabetes and thyroid dysfunction are found to subsist in chorus. Clinically overt disorders are considered only the tip of the autoimmune iceberg, since dormant forms are much more frequent [1]. There are three types of polyglandular autoimmune syndrome (PAS) including type I, type II and type III. Type II PAS, also known as Schmidt syndrome, is the most frequent PAS syndrome, which is usually found in concurrence with diabetes or thyroid disorders. The coexistence of thyroid dysfunction and diabetes have been discovered by many researchers [2–5]. Diabetic patients have susceptibility to different types of thyroid dysfunction, either hypothyroidism or hyperthyroidism, while patients with thyroid dysfunction are also susceptible to suffer from either type 1 diabetes or type 2 diabetes [6, 7]. Considering the strong connection between diabetes and thyroid diseases, the American Diabetes Association suggests that people with diabetes must be checked periodically for thyroid malfunction [8].

Male reproductive alterations have been extensively reported in diabetic individuals [9]. Hypothyroidism has been found to be more prevalent among diabetic population when compared with the normal population [10]. The blood-testis barrier (BTB) is a tight blood-tissue barrier that maintains adluminal environment and promotes spermatogenesis [11]. The effects of these concurrent metabolic pathologies on different systems of the body have been discussed only as retrospective studies on the basis of clinical case recorded in humans, while the data are lacking in the context of research trials for such syndromes and their effects on reproductive health. Claudins are mediators of the tight junction permeability and epithelial barrier function, and the tissue-specific barrier characteristics are hard to identify without determining the expression of claudin isoforms [12]. The trauma and any surgical intervention may damage the BTB, which leads to an autoimmune response of blood cells against the sperm. [13–16]. Although many researchers has examined the testicular cell development and sperm production of male animals under diverse disease conditions, however, the data are found lacking for the expression and immunolocalization of Claudin-11 in the testis of diabetes and hypothyroid mice. To determine the influence of diabetes combined with hypothyroidism on the male reproduction, we mimicked polyglandular complication and undertook a series of experiments in mice.

## Methods

### Experimental animals and treatments

Sixteen female ICR (Institute of Cancer Research) mice at day 15 of pregnancy were purchased from the Qinglongshan Laboratory Animal Company (Nanjing, China). These pregnant females were kept in the room with controlled temperature (21–22 °C), lighting (12-h light, 12-h dark). Before parturition each pregnant female was kept in separate cage. After parturition, mums along with their male pups were randomly assigned into four groups: control (C), diabetic (D), diabetic + hypothyroidism (Dh) and hypothyroidism (h). Each group of animals were comprising 2 to 3 mums and 12 to 15 male pups. STZ (streptozotocin, Cat. 18,883–66-4, Sigma-Aldrich, St Louis, MO, USA) was dissolved in the cold citrate buffer (Citric acid + Sodium citrate at 1:1.3 with pH 4.4) just before injection. Since spermatogenesis was found to start at the day of birth and the first A spermatogonia could be recognized at day 3 post partum in mice [17], the pups of group D and Dh received 3 intra-peritoneal injections of STZ (40 mg/kg bodyweight) on postnatal day 3, 4 and 8 [18] and the control received vehicle alone (placebo). The postpartum lactating females of groups Dh and h were offered 1-methyl-2-mercaptoimidazole, also known as Thiamazole (MMI) 0.05% + potassium perchlorate (KClO_4_) 0.5% in drinking water to induce pups hypothyroidism indirectly through milk feeding [19, 20]. After weaning (24 days) half of the male pups from each group were sacrificed and then remaining half continued to get the same treatment individually until 56 days old.

### Collection of samples

At postnatal day 24 (immature), six mice from each group anesthetized with halothane to measure the body weight and collect blood samples, and then they were euthanized by dislocating their neck. The left testis and epididymis were fixed in 4% (*w*/*v*) paraformaldehyde overnight and processed a regular way for histo-morphological analysis. The blood samples were centrifuged at 4000×g for 10 min to retrieve sera and stored at − 80 °C until further use.

Spermatozoa sample of mice (prepubertal) at 56 days old were collected as described in our previous study [21]. Cauda epididymis from all mice were carefully collected and washed with normal saline at 37 °C, then transferred to 1.5 ml tube containing 500μl artificial human tubular fluid (HTF; 37 °C) medium for recipe see [22]. After 5 min incubation at 37 °C in 5% CO_2_/95% air, the cauda epididymides were incised 5–7 times inside the tube and incubated for 15 min under the same condition to allow release of spermatozoa into the medium.

### Biochemical assays

During sacrificing, blood from orbital artery was used to check non-fasting blood glucose levels by using Sannuo rapid blood glucose meter (Sinocare Inc., Changsha, China), note that the results crossing the maximal limit (27.8 mmol/L) of the screening device were presented as 28 mmol/L. Serum concentrations of different hormones were determined by commercial radioimmunoassay (RIA) kits (North Institute of Biotechnology, Beijing, China) at the General Hospital of the Nanjing Military Command, Nanjing, China. The sensitivity determinations of insulin like growth factor 1 (IGF-1), testosterone (T), free thyroxine (fT4) and free triiodothyronine (fT3) were recorded < 5 ng/ml, 0.02 ng/ml, 1fmol/ml and 0.5fmol/ml, respectively. The intra-assay and inter-assay coefficients of variation for all these hormones (IGF1, T, fT4 and fT3) were < 10 and < 15%.

### Sperm counting and motility assessment

Sperm suspension medium (HTF) was diluted to 1:20, and average numbers of spermatozoa were counted by putting 10 μL sperm suspension on each side of a Neubauer chambered slide. Four large corner and the center squares were chosen to perform counting and the average sperm density was expressed in millions per millimeters.

Ten microliters of prepared sample was used for sperm motility assessment by Computer-assisted sperm analysis (CASA), in which 30 frames were analyzed in 0.5 s with six measurements of more than 2000 spermatozoa per animal.

### Histo-morphometric analyses

Fixed testis and epididymis tissue samples were dehydrated through a graded series of alcohol, cleared in xylene, and embedded in paraffin. The sections were cut at 5-μm thickness perpendicular to the longest axis of the tissues, mounted on glass slides, and stained with hematoxylin and eosin (HE). Histo-morphological changes were observed through a light microscope (Nikon, Tokyo, Japan) by three independent observers on request, which were unaware of the slide identity. Germ cells, epithelial cells and interstitial spaces were examined, with their diameters, extent of epithelial thickening and size of lumen of the tubules recorded in micrometers.

The morphometric measurements were done according to a systematic method of microscopic analysis [23], in which ten randomly selected sequential seminiferous tubules from each replicate sample were evaluated from one edge to the center of testes under 100×, 400×, and 1000× magnifications and the different measurements were recorded through microscopic calibration. Epididymal tubules were examined in the proximal caput and measurements were conducted horizontally from one edge to the next for all visible tubules. All apparent tubules were evaluated in the distal cauda region.

### Immunolocalization of oligodendrocyte-specific protein/Claudin-11 in mouse testes

Following deparaffinization and hydration of testicular sections in a successive series of xylene and ethanol, slides were then heated in 0.01 mol/L citrate buffer for 5–8 min in a microwave pressure cooker. The endogenous peroxidase activity and non-specific binding were blocked with 10% of H_2_O_2_ and bovine serum albumin (BSA, A4737, Sigma-Aldrich, St Louis, MO, USA) for 1 h, respectively. Slides were then incubated overnight at room temperature with Claudin-11 antibody (diluted 1:100). The immune reactivity of this specific protein was detected with rabbit IgG-SABC kits (SA1023/SA2002; Boster Biological Technology, Wuhan, China) and visualized with 0.05% 3, 3′-diaminobenzidine tetrachloride (E2; D8001; Sigma-Aldrich, St Louis, MO, USA) in 10 mmol/L PBS containing 0.01% H_2_O_2_ for 1–2 min. The negative control sections were incubated with PBS instead of the primary antibody. Finally, the reacted sections were counterstained with haematoxylin solution and mounted with cover slips, and the images were captured under a microscope (Nikon YS100; Nikon, Tokyo, Japan).

### Immunohistochemistry (IHC) quantification through digital image analysis

The measurement of DAB color intensity through their pixels, was done according to previous methodology [24]. The DAB and hematoxylin stained IHC digital images, captured at 400× magnification were used for analysis by ImageJ software. DAB, hematoxylin and a complimentary were produced by automating integrating deconvolution and histogram profiling where the scores and the number of pixels were calculated.

The digital image analysis requires standards of color pixel intensity values, which ranges from 0 to 255 (0 describes the darkest shade of the color and 255 depicts the lightest shade of the color). Since the expressions of Claudin-11 are low in between Sertoli cells, that is why this study computed the score of DAB color pixels by prescribed formula:

Score =$\frac{\mathrm{Number}\ \mathrm{of}\ \mathrm{pixels}\ \mathrm{in}\ \mathrm{a}\ \mathrm{zone}\times \mathrm{score}\ \mathrm{of}\ \mathrm{the}\ \mathrm{zone}\ }{\mathrm{Total}\ \mathrm{number}\ \mathrm{of}\ \mathrm{pixels}\ \mathrm{in}\ \mathrm{the}\ \mathrm{image}}$. A total number of 150 images were analyzed independently in the light of available scores with the assistance of two histo-pathological experts.

### Statistical analysis

Computations were carried out with SPSS (Version 17.0) and Graph Pad Prism (Version 5.0). All values were expressed as mean ± standard error of the mean (SEM). The differences across groups were calculated with one-way analysis of variance (ANOVA), followed by Tukey’s post hoc test and two-way ANOVA by considering Bonferroni posttests to compare the means of the replicates, where *P* < 0.05 was considered significant.

## Results

### STZ or/and MMI administration inhibits serum concentrations of IGF-1 and testosterone in mice

**Fig. 1:**
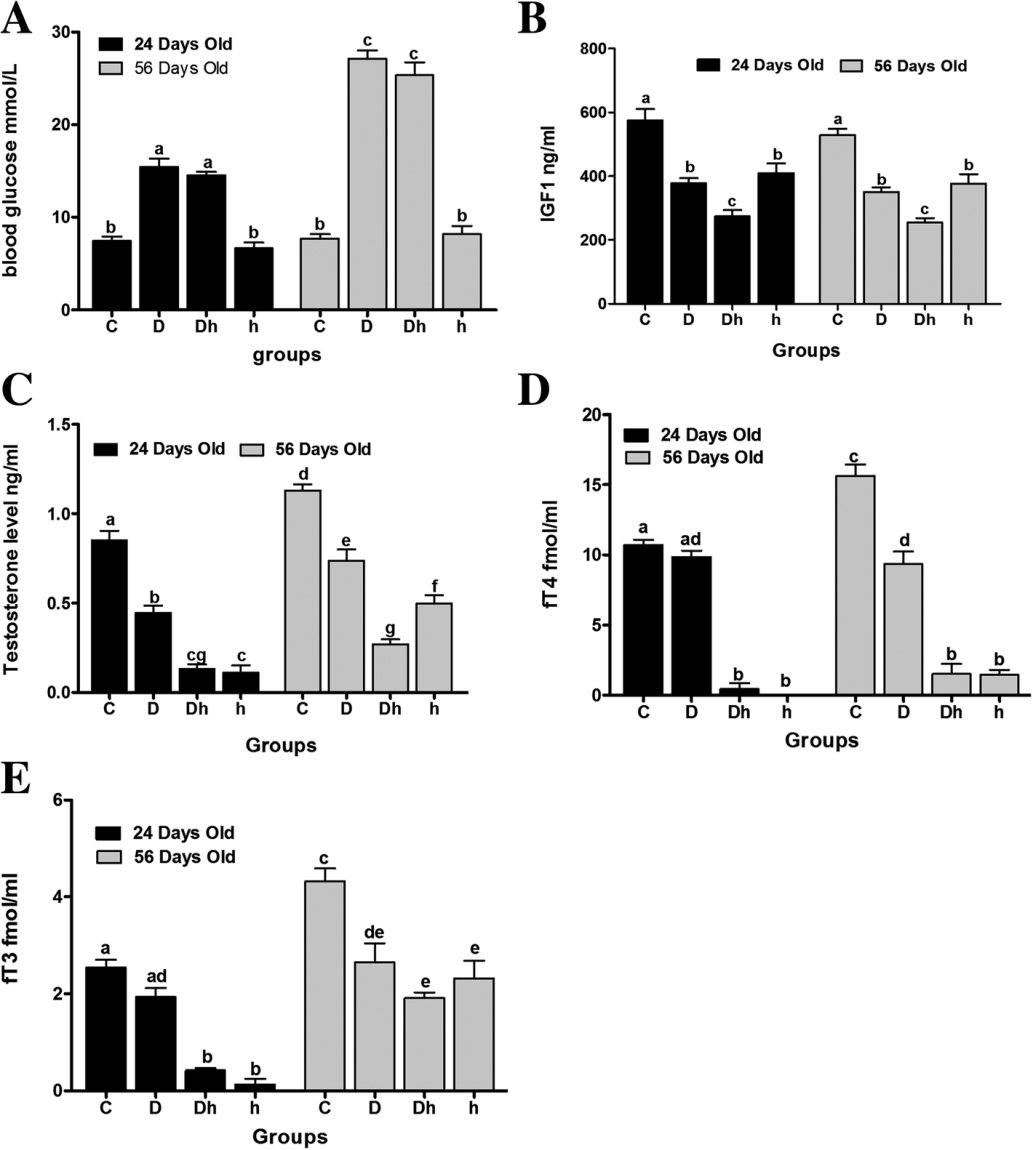
The blood glucose levels and concentrations of different measured hormones under the influence of diabetes mellitus and hypothyroidism. (A) Blood Glucose level, (B) IGF-1, (C) testosterone, (D) fT4 and (E) fT3. Data are represented as mean ± SEM (*n* = 6), and different labels indicated significant differences among groups at *P* < 0.05. Abbreviations: Control (C), Diabetic (D), diabetic + hypothyroidism (Dh) and Hypothyroidism (h)

To assess the effects of diabetes and hypothyroidism, we first established animal models of diabetic, diabetic plus hypothyroid and hypothyroid, treated with STZ, STZ + MMI, and MMI, respectively. The blood glucose was significantly increased in diabetic and diabetic plus hypothyroid groups in both immature and prepubertal mice after STZ treatment (Fig. 1A, Additional file 1). Serum concentrations of triiodothyronine / thyroxine (fT3/fT4) were decreased following MMI administration in diabetic plus hypothyroid and hypothyroid of both age group mice (Fig. 1D and E, Additional file 2). Experimental animals of diabetic and diabetic plus hypothyroid groups exhibited symptoms of polydypsia, polyphagia and polyuria throughout the trial.

Furthermore, we measured serum IGF-1 and testosterone (Additional file 2), which are the essential factors for testicular development, to investigate the influence of diabetes and hypothyroidism. We found serum IGF-1 levels were remarkably decreased after STZ or MMI treatment in both immature and prepubertal mice, which was even more diminished in the syndrome group (Fig. 1B). Serum testosterone levels of immature mice were also strongly inhibited after STZ, STZ + MMI or MMI administration, in which we barely detected lowered testosterone levels in Diabetic plus hypothyroid and hypothyroid groups. In prepubertal mice, serum concentrations of testosterone showed a similar inhibitory pattern in diabetic, diabetic plus hypothyroid groups, while mice in hypothyroid group had a higher testosterone levels than that of diabetic plus hypothyroid animals (Fig. 1C). Together, both diabetes and hypothyroidism may negatively regulate testicular development by inhibiting IGF-1 and testosterone.

### Reduction of serum IGF-1 and testosterone levels decreases the body weight, testes weight and epididymal weight in mice

**Fig. 2:**
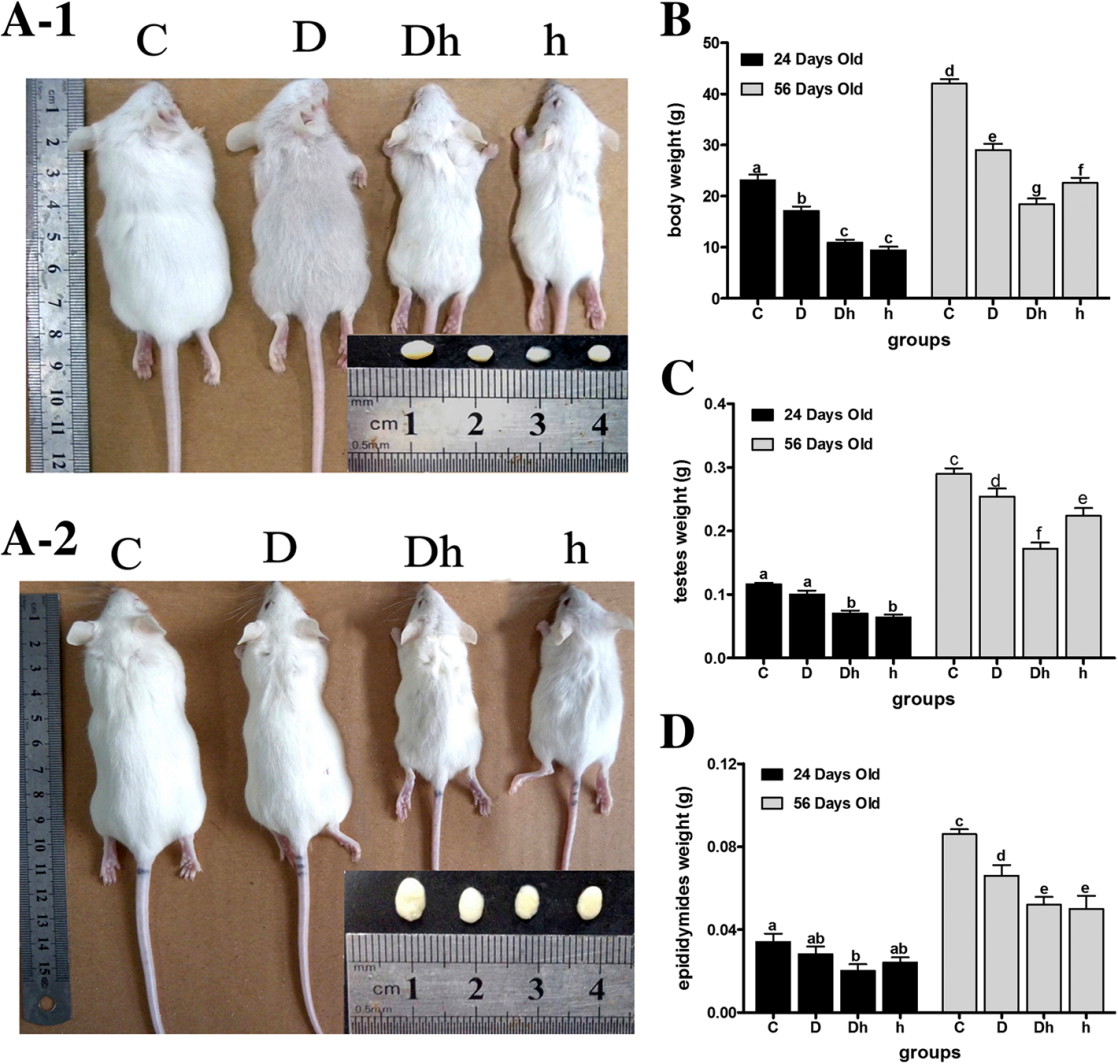
Effects of STZ-diabetes and hypothyroidism on body, testes and epididymides weights in immature- to prepubertal mice. (A) Photographs of mice along with their testes at the age of 24 days old (top panel) and 56 days old (bottom panel). (B) Body weight, (C) testes weight and (D) epididymides weight under the influence of diabetes and hypothyroidism conditions. Data are represented mean ± SEM (n = 6), and different labels indicated significant differences among groups at *P* < 0.05. Abbreviations: Control (C), Diabetic (D), diabetic + hypothyroidism (Dh) and Hypothyroidism (h)

Since IGF-1 and testosterone are essential for development, we then tested whether contemporaneous diabetes with hypothyroidism influence the body weight, testes weight and epididymal weight or not (Additional file 1). In prepubertal mice, the weight of body, testes and epididymides decreased by 31, 12 and 23% under diabetes condition, and by 46, 23 and 42%, respectively in hypothyroid mice. The body and testes weights were even further decreased by 56 and 41%, respectively in syndrome group of mice, which were suffering from both, diabetes plus hypothyroidism than any of the each condition alone (Fig. 2 B and C). Similarly the immature mice body weight decreased by 26 and 60% in diabetes and hypothyroid mice, respectively. Hypothyroidism inhibited testes weight of immature mice by 40%. Both hypothyroidism and diabetes showed no effects on epididymal weights of immature mice. However, significantly decreased body and testes weights were observed in the mice, suffering from the diabetes plus hypothyroidism condition (Fig. 2 B and C). Our data indicated that the contemporaneous diabetes with hypothyroidism had adverse effects on the testicular architecture and sperm parameters of the mice.

### Contemporaneous diabetes with hypothyroidism damages testicular and epididymal morphology

**Fig. 3:**
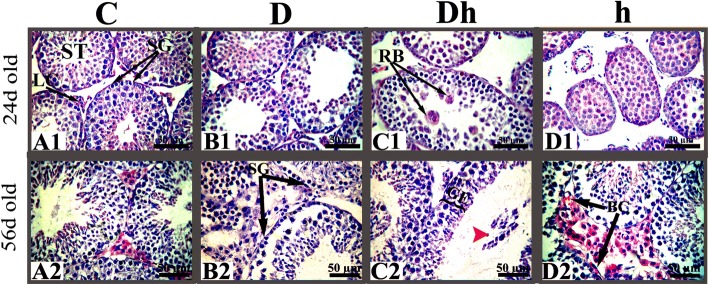
Histo-architectural changes in testicular cells during experimental diabetes mellitus and hypothyroidism in immature and prepubertal mice. Sloughed spermatids, marked with red arrow head shown inside the lumen of tubule of Dh animals (panel: C2). Abbreviations: Control (C), Diabetic (D), diabetic + hypothyroidism (Dh) and Hypothyroidism (h), Seminiferous tubule (ST), Blood cells (BC), Leydig cells (LC), Spermatogonia (SG), Germinal epithelium (GE),. Representative images were captured at 400× magnifications. The bars are 50 μm in size

To evaluate the potential negative effect caused by contemporaneous diabetes with hypothyroidism, we first analyzed the testicular morphological changes caused by diabetes, hypothyroidism and diabetes plus hypothyroidism (Additional file 3). We found that hypothyroidism inhibited seminiferous tubules development in both immature and prepubertal mice by reducing its diameter by 36 and 19%, respectively compared with control. While, the luminal size of these seminiferous tubules were increased in diabetic mice by 62 and 31% in immature and prepubertal mice, respectively (Table 1). Although seminiferous tubule lumen size of Diabetic plus hypothyroid of immature mice showed increased (19%) values with no statistical difference from control animals, while significantly increased (31%) at the age of 56 days (Table 1). These results suggested that diabetes was unable to rescue hypothyroidism induced seminiferous tubules developmental dysfunction, because we found numbers of residual bodies in the seminiferous tubule lumen of immature diabetic plus hypothyroid mice (Fig. 3). In prepubertal diabetic plus hypothyroid mice, the size of seminiferous tubule and epithelium were smaller by 15 and 20%, respectively, compared with the control. (Table 1). We also found sloughed spermatids in many of the seminiferous tubule lumens of diabetic plus hypothyroid mice (Fig. 3), which was indicator of testicular dysplasia.

**Table 1:** Microscopic calibrated measurements of different parts of seminiferous and epididymal tubules in micro meters (μm), during different age levels

Age Level	Groups	St diameter	St Lumen diameter	St Epithelial height	Caput diameter	Caput lumen diameter	Caput Epithelial height	Cauda diameter	Cauda lumen diameter	Cauda Epithelial height
24 days old	Control	146.2 ± 2.3^a^	33.5 ± 2.5^bc^	55.4 ± 1.4^a^	89.4 ± 2.3^a^	35.5 ± 1.2^a^	23.6 ± 0.7^a^	122.1 ± 3.3^a^	60.9 ± 2.6^a^	35.9 ± 1.3^a^
	Diabetic	128.7 ± 1.9^b^	54.4 ± 2.3^a^	41.1 ± 1.2^b^	64.4 ± 1.9^b^	23.3 ± 0.8^b^	21.9 ± 0.6^a^	110.8 ± 3.5^a^	41.8 ± 3.1^b^	36.7 ± 1.3^a^
	Diabetic + Hypo	122.3 ± 1.2^b^	39.9 ± 1.6^b^	40.6 ± 1.4^b^	61.7 ± 2.5^b^	22.7 ± 1.5^b^	18.6 ± 0.9^b^	56.1 ± 3.2^b^	19.2 ± 1.1^c^	24.9 ± 1.0^b^
	Hypo	93.9 ± 2.3^c^	24.6 ± 1.1^c^	29.8 ± 1.3^c^	49.6 ± 1.1^c^	26.3 ± 0.8^b^	14.7 ± 0.5^c^	66.9 ± 3.1^b^	33.4 ± 2.3^b^	17.2 ± 1.0^c^
56 days old	Control	205.3 ± 5.0^d^	63.9 ± 3.3^e^	69.9 ± 1.5^d^	129.1 ± 2.6^d^	68.0 ± 1.4^c^	28.0 ± 1.3^d^	248.2 ± 6.2^c^	207.8 ± 8.3^d^	10.9 ± 0.9^e^
	Diabetic	198.6 ± 4.3^d^	83.9 ± 2.4^d^	57.0 ± 2.2^e^	114.8 ± 2.7^e^	68.5 ± 1.6^c^	27.9 ± 0.9^d^	207.0 ± 6.5^d^	183.2 ± 5.6^e^	14.3 ± 0.8^d^
	Diabetic + Hypo	174.5 ± 2.7^e^	86.4 ± 5.0^d^	56.1 ± 2.3^e^	88.5 ± 1.0^f^	53.3 ± 1.0^d^	17.9 ± 0.3^b^	135.1 ± 5.4^f^	106.2 ± 2.3^g^	16.2 ± 0.7^d^
	Hypo	166.4 ± 4.6^e^	63.0 ± 2.6^e^	68.8 ± 2.7^d^	116.3 ± 1.9^e^	66.9 ± 1.4^c^	27.3 ± 1.0^d^	158.0 ± 6.0^e^	121.6 ± 2.7^f^	15.7 ± 0.6^cd^

Data are presented as mean ± SEM (n = 6) and different labeled letters indicated significant differences among groups at *P* < 0.05

Abbreviations: Hypothyroidism (hypo) , seminiferous tubule (St)

**Fig. 4:**
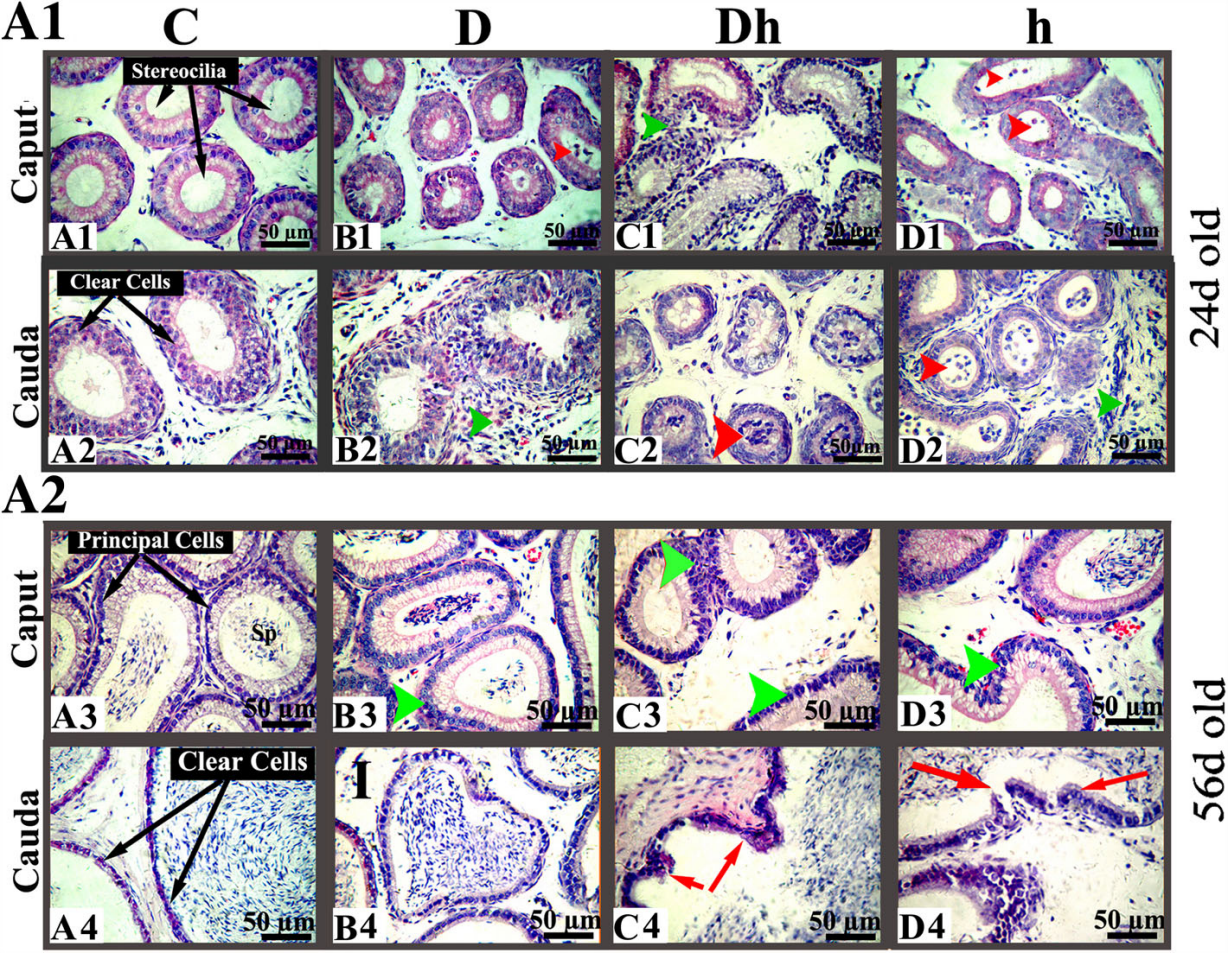
Hematoxylin and eosin stained digital images of the epididymis at different age levels of mice, showing proximal caput and distal cauda under the influence of diabetes and hypothyroidism compared with control. **(A1)** Epididymal sections of immature mice at the age of 24 days old. Most of the epididymal tubules of treated animals possess round bodies with exfoliated germ cells, marked with red arrow heads (panels B1, D1, C2 and D2). Some of the epididymal tubules of treated mice, depicts inflammatory infiltrations marked with green arrow heads (panels C1, B2 and D2). **(A2)** Epididymal sections of pre pubertal mice at the age of 56 days old presenting hyperplastic changes in principal cells of caput tubules are shown with green arrow heads (panels: B3, C3 and D3). Cribriform changes are marked with red arrows (pannels: C4 and D4). Abbreviations: Control (C), Diabetic (D), diabetic + hypothyroidism (Dh) and Hypothyroidism (h), (S) Spermatozoa and (I) Interstitium. The pictures were captured at magnification of 400× and the bars are 50 μm in size

We determined the morphological changes in epididymis caused by contemporaneous diabetes plus hypothyroidism in immature mice (Additional file 3). Herein, we found well organized principle cells and stereocilia only in caput epididymis of control mice. Whereas the caput tubules of diabetic or hypothyroid mice were not well developed (Fig. 4 A1). The diameter of caput tubules were also smaller in Diabetic (28%), Diabetic plus hypothyroid (31%) and hypothyroid (45%) mice compared with control (Table 1), with less differentiated epithelial structures and presence of exfoliated germ cells (Fig. 4 A1 marked with red arrow). Furthermore, we observed inflammatory infiltrations in caput epididymis of diabetic plus hypothyroid and hypothyroid mice (Fig. 4 A1 marked as green arrow), which results in sperm death and a loss of spermatogenic function. In cauda epididymis, tubules were smaller in hypothyroid (45%) and diabetic plus hypothyroid (54%) mice comparing with control animals (Table 1). These results indicated that hypothyroidism induced inhibition of epididymal development. We also found exfoliated germ cells existed in the hypothyroid and diabetic plus hypothyroid mice epididymis (Fig. 4A marked with red arrow). Inflammatory infiltrations existed in cauda lumen of diabetic mice (Fig. 4 A1).

To further investigate the influence of diabetes and hypothyroidism to prepubertal mice reproduction, we analyzed the morphological changes in the epididymis of diabetic, diabetic plus hypothyroid and hypothyroid mice (Additional file 3). We found hyperplastic changes in principal cells of caput tubules in diabetic, diabetic plus hypothyroid and hypothyroid mice (Fig. 4 A2), though there was less change in their size (Table 1). However, sperms existed only in the caput tubules of control mice and diabetic mice, whereas only less than half of the caput tubules contained spermatozoa in diabetic plus hypothyroid and hypothyroid groups (Fig. 4 A2). Furthermore, there was more space between caput tubules of diabetic plus hypothyroid and hypothyroid mice compared with control and diabetic mice. We found thin and healthy cauda epithelia inside the tubules of control mice, while the epithelia were thicker in diabetic, diabetic plus hypothyroid and hypothyroid by 31, 49 and 44%, respectively. Despite epithelia, the cauda tubules were smaller in diabetic (17%), diabetic plus hypothyroid (46%) and hypothyroid (36%) mice (Fig. 4 A2 and Table 1). We also found inflammatory infiltrations in the epithelia of these tubules together with cribriform changes in the diabetic plus hypothyroid and hypothyroid (Fig. 4 A2). Our results indicated that contemporaneous diabetes combined with hypothyroidism adversely affected the process of spermatogenesis.

### Claudin-11 is expressed low in BTB of contemporaneous diabetes combined with hypothyroidism mice

**Fig. 5:**
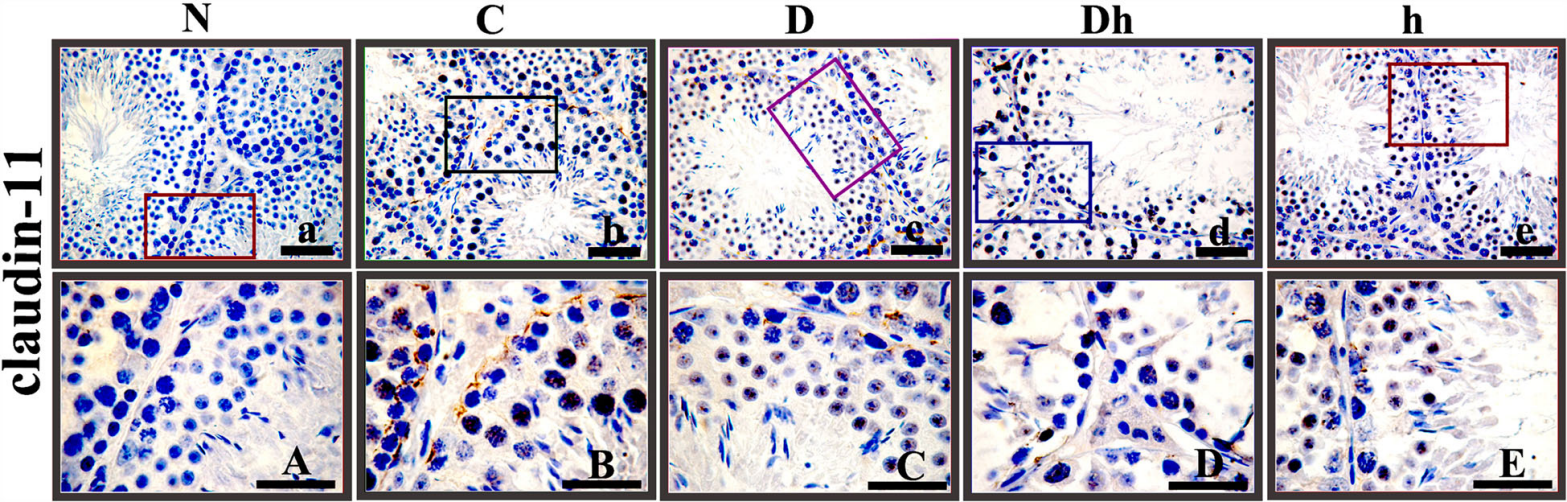
Immunolocalization of the Claudin-11 in the testes of adult mice, exposed to experimental diabetes mellitus and hypothyroidism. Immunostaining was seen positive with DAB brown along with blue counter stained with hematoxylin. Significantly either week (low positive) or disrupted expressions of Claudin-11 were noticed in syndrome group mice (Dh), followed by h and D compared with control. These pictures were captured at magnification of 400× and 1000× with pasted bars of 50 and 30 μm in size at the top and bottom rows, respectively

**Fig. 6:**
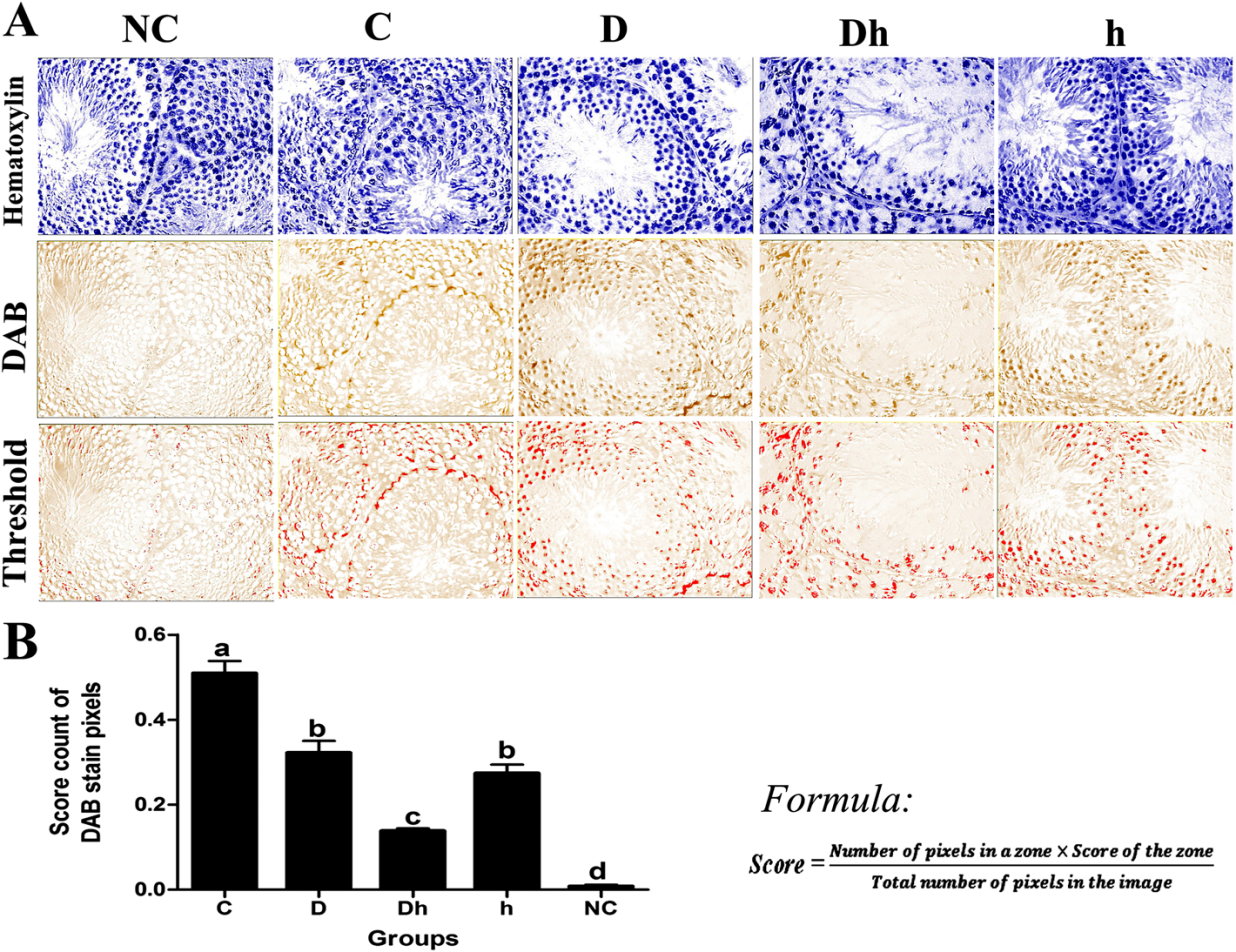
Representative digital images of histogram profile shows DAB brown staining, color pixel intensity for Claudin-11 in the testes of adult mice, exposed to experimental diabetes mellitus and hypothyroidism. **(A)** From top to bottom rows; panels shows the digital image masks stained with hematoxylin, DAB and threshold respectively. **(B)** Claudin-11 is expressed in a limited quantity at tight junction in between Sertoli cells of all treated groups that is why analyzed through score calculation. Data are representing mean ± SEM (*n* = 6) and different labels indicated significant differences among groups at *P* < 0.05

The tight junction of blood testes barrier (BTB) is essential in testicular development and spermatogenesis. To assess the influence of diabetes and hypothyroidism on the maintenance of this junction, we detected the localization of Claudin-11 in the testes through immunohistochemistry (IHC). By using a specific antibody, we found Claudin-11 expressed in Sertoli cells of control mice, by forming a regular zigzag border like structure around seminiferous epithelium (Fig. 5). Comparing with control mice, we found weak staining of the Claudin-11 in Sertoli cells of diabetic, diabetic plus hypothyroid and hypothyroid mice. To further evaluate the effect of hypothyroidism and diabetes on the tight junction of BTB, we performed a quantitative analysis of the Claudin-11 protein in the IHC sections. The expressions of Claudin-11 inside the seminiferous tubules were decreased by 46 and 37% in hypothyroid and diabetic mice, respectively, while it was strongly repressed by 73% in Diabetic plus hypothyroid group of animals (Fig. 6B). These data indicated that contemporaneous diabetes with hypothyroidism severely damaged the structure of tight junction of BTB inside the seminiferous tubules.

### Diabetes combined with hypothyroidism inhibits spermatogenesis and decreased sperm motility

**Fig. 7:**
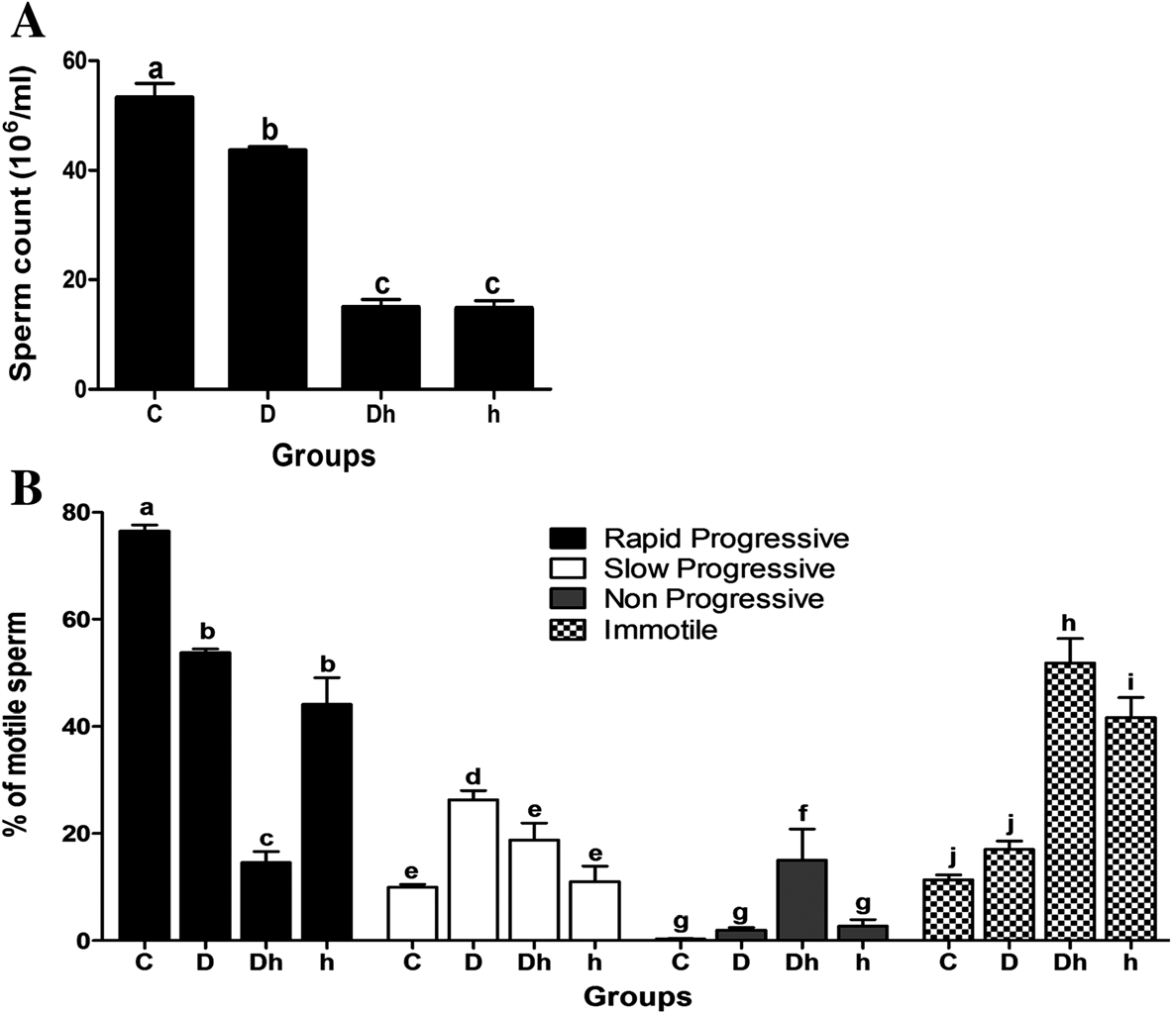
Sperm concentration and motility of diabetic and hypothyroid mice during the age of 08 weeks. **(A)** Decreased sperm count was critically noticed in Dh and h group of animals compared to D and C. **(B)** Decreased rapid progressive motion of spermatozoa was recorded in all treated mice compared with control. However, sperm motility parameters were critically reduced in Dh subjects. Data represented mean ± SEM (n = 6) and different labels indicated significant differences among groups at *P* < 0.05

To address whether contemporaneous diabetes combined with hypothyroidism influence spermatogenesis or not, we first isolated fresh sperm from cauda. Then by using a classic method, we counted the number of sperm from all experimental groups (Additional file 4). We found that diabetes only slightly decreased total sperm number (10%), while hypothyroidism strongly reduced the number of sperm (75%). Hence, contemporaneous diabetes with hypothyroidism also significantly decreased the sperm count by 75% compared with control (Fig. 7A). Then, using a sperm motility analysis system, we further investigated the sperm quality changes caused by diabetes and hypothyroidism. We found the proportion of rapid progressive sperms decreased by 30~ 40% in diabetic and hypothyroid mice, while only 19% rapid progressive sperm existed in diabetic plus hypothyroid mice. In contrast, the proportion of immotile spermatozoa were found highest in number in diabetic plus hypothyroid mice by comparing with other three groups (Fig. 7B). The sperm from diabetes mice also showed the slow progression of sperm motility (Fig. 7B). Our data demonstrated that contemporaneous diabetes with hypothyroidism not only reduced the number of sperm, but also seriously inhibited sperm motility.

## Discussion

Diabetes and hypothyroidism produce adverse effects on the body weight and sexual maturity by inhibiting body growth and metabolism [25, 26]. The occurrence of diabetes is always accompanied with thyroid dysfunction. Thus, it is important to take hypo- or hyper-thyroidism into consideration when exploring the adverse effects caused by diabetes. Previous reports have found hypothyroidism inhibits testicular growth by delaying Sertoli cell differentiation and proliferation [27]. Hence, by establishing a mouse model of diabetes combined with hypothyroidism, we provided evidence that poly glandular autoimmune syndrome affected testicular development and spermatogenesis. These data demonstrated that diabetes combined with hypothyroidism inhibited serum IGF-1 and testosterone synthesis. Moreover, IGF-1 and testosterone inhibited the development of testicular and epididymal tissues. Our data also suggested that the impaired and/or reduced expressions of the Claudin-11 caused the damage of BTB. The inhibitory effects to testicular and epididymal development along with BTB deficiency resulted in sperm number reduction and decreased sperm quality. To our knowledge, this is the first study to mimic mice poly glandular autoimmune syndrome and to investigate their effects on the male reproduction.

Similar to previous reports of rat hypothyroidism, we found reduced testicular weights of both immature and prepubertal mice following concomitant induction of diabetes and hypothyroidism. Compared with short term treatment, diabetic mice has been reported to show a reduction in testicular weights only in long term study [20, 27]. Consistent with previous studies, we found no statistical differences in epididymal weights between control and hypothyroid mice during the age of 24 days [20]. However, we found epididymal weights reduced in the pre-pubertal mice of syndrome group (Dh). Our data indicated a combined inhibition to male sexual organ development caused by hypothyroidism and diabetes.

The insulin-like growth factors (IGFs) are proteins with high similarity to insulin. It is well identified that insulin/IGF signaling pathway is essential for FSH-mediated Sertoli cell proliferation and testicular development [9, 28]. IGF-1, a 70 amino acid protein, is essential in stimulating cell growth and development. Consistent to several clinical studies of diabetic and hypothyroid patients, we found the serum level of IGF-1 remarkably decreased in the diabetic or hypothyroid mice in comparison to control group, however, it was found critically lower in syndrome group of mice [29, 30].

IGF-1 is also an important growth factor modulating testosterone biosynthesis [31–34]. Thus, the decrease of serum IGF-1 levels will result in serum testosterone reduction. In this study, we found serum testosterone, an important hormone regulating testicular development, decreased in diabetic and hypothyroid mice [9, 20, 35, 36]. Thyroid hormones, especially T3, have been reported as key factor in production of testosterone from Leydig cells and increase LH receptor expression [37]. Hence, we found hypothyroid mice revealed a lower serum testosterone levels, as well as diabetes combined with hypothyroidism mice. Collectively, we suggest that diabetes combined with hypothyroidism affects testicular development by regulating IGF-1/testosterone signaling. Our data also indicated that thyroid hormones dominantly regulated testosterone and testicular development in diabetic cum hypothyroid mice.

Previous reports have demonstrated that diabetes or hypothyroidism alone is able to inhibit testicular development [38–41]. Diabetes, especially type I, disrupts the hormone homeostasis of hypothalamic pituitary gonadal axis, and consequently results in testicular histological changes, Leydig cells shrinking and spermatogenesis dysfunction in male animals [35]. Similarly, hypothyroidism leads to a decrease in serum LH and FSH resulting in small testes size, decelerated Sertoli cell differentiation and prolonged their proliferation in rats [27, 42, 43]. Collectively, we propose that diabetes and hypothyroidism together can decisively inhibit the growth and development of testicular and epididymal tissues.

Consistent to the hypothesis we proposed, our data demonstrated that diabetes combined with hypothyroidism disrupted testicular and epididymal growth in both immature and prepubertal mice. The morphology of testes and epididymis reflected their developmental status. However, our study suggested that autoimmune diseases were chronic in nature, it was therefore longer studies periods were being recommended for future observations from neonatal to adult and old age animals.

**In our study, the control mice showed well organized** columnar cells (principal cells), basal cells, and clear cells in these tubules. The concomitant diabetes mellitus-plus-hypothyroidism critically damaged the **ductus efferentes** and the **ductus epididymis** in both immature and prepubertal mice. The tall columnar epithelial cells became thin or possess damaged stereocilia in diabetic cum hypothyroid mice. We also found inflammatory infiltrations in most of the epididymal tubules of all treated mice, indicating distortion of the BTB. In immature mice, exfoliated germ cells and rough round bodies existed in caput and cauda of diabetes combined with hypothyroidism or hypothyroid mice. Moreover, we found few spermatozoa in the lumen of prepubertal Dh or h mice, followed by increased interstitial stroma, dispersed red blood cells, lipid vacuolization and cribriform/hyperplasia. Our data indicate that diabetes combined with hypothyroidism inhibits mice spermatogenesis.

The basic morphologic and physiologic conditions of epididymis are required for successful sperm transport and fertilizing capacity [44]. The epididymis, an important store house of spermatozoa, can be affected by the direct and indirect disorders of the testis. Previous reports have discovered histological changes in pre-pubertal rats with STZ-induced diabetes [45, 46], in which the expression of androgen-binding protein decrease in epididymis [47]. Similarly, the epididymis of Albino rats exhibited diminished testosterone level, androgen-binding protein, sialic acid, glyceryl phosphorylcholine, and carnitine suggesting its detrimental effects on the epithelial physio-morphology [48]. Moreover, sperm count, progressive motility and DNA integrity are found decreased in STZ-induced diabetic mice [49]. In epididymis, thyroid hormones bind to its receptors in both the nuclear and cytoplasmic compartment of epithelial cells [50]. Thyroid hormone deficiency adversely affects the sperm morphology and progressive motility [51]. A clinic research driven from 66 individuals reveals that the sperm count, motility, morphology and erectile function decrease in hypothyroid patients [52]. Thus, diabetes combined with hypothyroidism may result in reproduction dysfunction by influencing sperm quality.

Consistent with previous reports, we demonstrate that diabetes or hypothyroidism causes a reduction in sperm count and sperm quality. Our data suggest that hypothyroidism dominantly reduces sperm count in diabetes combined with hypothyroidism mice. Moreover, diabetes or hypothyroidism only slightly decreases progressive sperm motility. While the proportion of immotile or non-progressive sperm was the highest in syndrome group. Therefore, the disorder of testicular and epididymal development caused by diabetes combined with hypothyroidism may result in decrease of sperm motility.

The germinal epithelia of seminiferous tubules are composed of a basal and an adluminal compartments. The adluminal compartment is engaged in meiosis and spermatogenesis, whereas the renewal and proliferation of spermatogonia occurs in the basal compartment [53, 54]. The BTB is a barrier between blood vessels and the seminiferous tubules of the animal testis, which is formed by tight, adherens and gap junctions between the Sertoli cells. The presence of the BTB helps Sertoli cells to modulate adluminal environment and prevent passage of cytotoxic agents into the seminiferous tubules. Claudin-11, a protein of tight junction, has a typical role in establishing the hemato-testicular barrier between the basal and adluminal compartments [55, 56]. Previous reports have revealed that high glucose levels have inhibitory effect on Claudin-5 and -11 in diabetic patients [57]. In this study, we found Claudin-11 expressed in the BTB between Sertoli cells. Consistent with previous reports, we found decreased expressions of Claudin-11 in the testes of diabetic and hypothyroid mice. While its expressions were seen significantly low in syndrome group mice. The loss of Claudin-11expression results in distortion of the BTB, which allows infiltrated immune cells to enter testicular tubules and kill spermatids. The existence of infiltrated immune cells also indicates that the BTB has lost its function to protect the spermatids inside the seminiferous tubules.

## Conclusion

In this study, we have provided the evidence that metabolic dysfunctions can produce distinct influence on the development of testes and epididymis through IGF-1 and testosterone, which further influence spermatogenesis and sperm motility under the involvement of Claudin-11. By evaluating the sperm quality and morphological changes in testes, our data provided new insight into the effects of type II PAS to male sexual organ development and spermatogenesis.

## Additional files

Additional file 1: Body weights, organ weights and blood glucose levels in mice on days 24 and 56 day. Data were shown as two sets for days 24 and 56, respectively. (XLSX 13 kb)

Additional file 2: IGF-1 and hormonal profiles at different ages of mice. Data were shown as IGF-1 and different hormones, separatly. (XLSX 12 kb)

Additional file 3: Micro Measurements of mice at days 24 and 56. Data were shown as diameters of the Seminiferous tubule and diferent parts of the Epididmis. (XLSX 22 kb)

Additional file 4: Sperm parameters. Data were shown each original data. (XLSX 9 kb)

## Abbreviations

BSA: Bovine Serum Albumin
BTB: Blood-testis-barrier
CASA: Computer assisted Sperm analysis
CdE: Cauda Epididymis
CpE: Caput Epididymis
DAB: 3,3′-diaminobenzidine etrahydrochloride
DM: Diabetes Mellitus
FSH: Follicle Stimulating Hormone
fT_3_: Free triiodothyronine
fT_4_: Free thyroxine
GnRH: Gonadotropin releasing hormone
ICR mice: Institute of Cancer Research mice
IHC: Immuno-histochemistry
KCLO_4_: Potassium perchlorate
LC: Leydig cell
LH: Luteinizing Hormone
LT_4_: Levothyroxine
MMI: Methylmercaptoimidozole
OSP: Oligodendrocyte-specific protein
PBS: Phosphate Buffered Saline
PS: Primary Spermatocyte
RIA: Radioimmunoassay
SABC: Strept Avidin Biotin Complex
SC: Sertoli Cell
SG: Spermatogonia
St: Seminiferous tubule
STZ: Streptozotocin
T: Testosterone
T1DM: Type-1 Diabetes Mellitus
T_3_: Triiodothyronine
T_4_: Thyroxine (3,3′,5,5′-tetraiodothyronine)
TD: Thyroid dysfunction
